# The Medical Student's Practical and Theoretical Guide to the Translation and Composition of Latin Prescriptions; with an Explanatory Latin Grammar, Written in a Familiar Style; Together with a Text-Book and Exercises, Calculated to Make the Learner Thoroughly Acquainted with the Verbal and Grammatical Analysis of the Pharmacopœia, Prescriptions, &c.

**Published:** 1836-04

**Authors:** 


Art. X.—*The Medical Student's Practical and Theoretical Guide to the Translation and Composition of Latin Prescriptions; with an Explanatory Latin Grammar, written in a familiar style; together with a Text-book and Exercises, calculated to make the Learner thoroughly acquainted with the verbal and grammatical Analysis of the Pharmacopœia, Prescriptions, &c.* By J. W. Underwood.—*London,* 1835. 12mo. pp. 132.

We looked into this little book, as in duty bound, firstly, because the author had the courtesy to send it to us; secondly, because (although hardly coming within the scope, ample as it is, announced in our title-page,) it might yet be of no small importance to a class of readers among whom we hope to find many abettors, and whose interests we shall ever regard as placed peculiarly under the vigilant eye of the directors of the periodical medical press. The student requires to be protected quite as much against the evil effects of his own well-designed miscalculations, and the counsels of well-meaning but injudicious friends, as against the operation of vexatious legal enactments, or ill-considered corporate regulations. We have not time at present to enter upon the subject of the preliminary education requisite for the medical student, of which the study of the Latin language constitutes so important a part; but we must be permitted to observe, in passing, that, if the recent regulation adopted by the Apothecaries' Company, of admitting young men to their classical examination on *commencing* their studies in London, be designed to exclude all further testing of the candidate's proficiency in the Latin language, when they present themselves for the licence after completing their medical studies, the rule is, in our opinion, most injudicious, and calculated to perpetuate and increase the very evils it was intended to mitigate or remove. It is surely not merely desirable that the student should give evidence that he has acquired a competent knowledge of the Latin language, previously to entering on the serious business of studies more strictly professional, but also that he preserves this knowledge during the progress of these studies, and carries it into practice with him. It would, no doubt, be of advantage to a medical student to have undergone the intellectual training implied by the study of the ancient languages, even if, on entering upon his profession, he were to lose all practical knowledge of them; but surely it will be equally admitted that the preservation of the knowledge acquired in the classical school-room, must be at least of as great importance to the practitioner of medicine. Now, does not the regulation alluded to almost seem to imply—(it being assumed that this preliminary examination is the *only* one in classics,) that the student, having once exhibited the requisite degree of competency in Latin, may for ever after disregard it? Or, at least, will not this be, in fact, the practical operation of the regulation on the minds of a considerable number of students? The learned directors of the Apothecaries' Company may have had a truer and more disinterested love of Latin for itself, and a more astringent memory for vocables and syntax, than we could ever boast of; but, for our own parts, we are bound, by the faith of honest criticism, although with shame and confusion of face, to acknowledge that, had it not been for the consciousness ever present to us during our medical studies, that we should have to *make our appearance in Latin* at the end of them, we might, in our free zeal for *science*, have actually forgot, if not all, at least much of the *literature* that had been forced upon us in our earlier years. We shall heartily rejoice to find that there is a difference in this respect between the students of present and former times, and that the good intentions of the Apothecaries' Company, which we do not for one moment call in question, will be fulfilled and our fears proved to be without foundation : we, however, to say the least, entertain very strong doubts on the subject.

Of the little work before us we would say, that, if any student, not well-grounded in the principles of the Latin language, imagines that, by means of it, he will be enabled to acquire any knowledge of that language that will be really useful to him in the study and practice of his profession, he will be utterly disappointed on making the trial. It is totally inadequate to fulfil so large a scope; and, indeed, no book can do so. As a grammar, it is correct as far as it goes, but it goes a very short way indeed. It does not include a tenth part of the essential rules of syntax; and a student, complete master of every one contained in it, and of none besides, would be confounded by the very first sentence he might be desired to construe and parse, in Celsus, Gregory, or the Pharmacopœia.

It would not, however, be doing justice to the author of this little book, if we did not state that he is evidently a good scholar himself, and that in general, he very successfully eschews, in his "Exercises," that species of Latinity which is too common, we fear, in professional practice, and which has been wittily said to have derived its stock of words from that part of the dictionary which used, in our school-days, to be entitled "Index vocum, ab iis qui Latine scribere velint, vitandarum." Celsus, Heberden, and Gregory, are evidently his authorities; and we can safely recommend his "*Key to the Prescriptions*," in the latter part of the volume, as containing a good collection of medical phrases suited for extemporaneous prescription, and to which even the classical student may apply with advantage, as containing phrases and vocables which may not be familiar, and yet very necessary to him.

